# Possible Contribution of Zerumbone-Induced Proteo-Stress to Its Anti-Inflammatory Functions via the Activation of Heat Shock Factor 1

**DOI:** 10.1371/journal.pone.0161282

**Published:** 2016-08-18

**Authors:** Yoko Igarashi, Kohta Ohnishi, Kazuhiro Irie, Akira Murakami

**Affiliations:** Division of Food Science and Biotechnology, Graduate School of Agriculture, Kyoto University, Kyoto, Japan; Boston University Medical School, UNITED STATES

## Abstract

Zerumbone is a sesquiterpene present in *Zinger zerumbet*. Many studies have demonstrated its marked anti-inflammatory and anti-carcinogenesis activities. Recently, we showed that zerumbone binds to numerous proteins with scant selectivity and induces the expression of heat shock proteins (HSPs) in hepatocytes. To dampen proteo-toxic stress, organisms have a stress-responsive molecular machinery, known as heat shock response. Heat shock factor 1 (HSF1) plays a key role in this protein quality control system by promoting activation of HSPs. In this study, we investigated whether zerumbone-induced HSF1 activation contributes to its anti-inflammatory functions in stimulated macrophages. Our findings showed that zerumbone increased cellular protein aggregates and promoted nuclear translocation of HSF1 for HSP expression. Interestingly, HSF1 down-regulation attenuated the suppressive effects of zerumbone on mRNA and protein expressions of pro-inflammatory genes, including inducible nitric oxide synthase and interlukin-1β. These results suggest that proteo-stress induced by zerumbone activates HSF1 for exhibiting its anti-inflammatory functions.

## Introduction

Zerumbone is a sesquiterpenoid present in the subtropical ginger *Zingiber zerumbet* Smith, a plant that has been traditionally utilized in Southeast Asia for various purposes, including anti-inflammatory medicine. Recent *in vitro* and *in vivo* studies have revealed several biological properties of this phytochemical, *e*.*g*., inhibitory effects toward free radical generation, expression of pro-inflammatory genes, such as inducible nitric oxide synthase (iNOS) and cyclooxygenase-2 (COX-2), and carcinogenesis [1–7]. Although the underlying molecular mechanisms of its cancer preventive activity have not been fully elucidated, we previously reported that zerumbone activated nuclear factor erythroid 2-related factor-2 (Nrf2) presumably by binding to Kelch-like ECH-associated protein 1 (Keap1), the key regulator of Nrf2 activation [8,9]. This binding may occur at its α,β-unsaturated carbonyl group, which can react with a nucleophilic group. On the other hand, our experiments using antibodies (Abs) against zerumbone-adducts showed that it binds to various cellular proteins with low selectivity [10]. Because treatment of cellular proteins *in vitro* with zerumbone leads to their insolubilization [10,11], such non-specific protein modifications are expected to cause proteo-toxic stress in cells.

Denatured proteins tend to form aggregates, in which their surface-exposed hydrophobic domains interact with each other, and accumulation of these abnormal proteins is related to onset of various conditions such as neurodegenerative disease [12]. In organisms, there are mechanisms to maintain protein homeostasis and prevent accumulation of protein aggregates, which are collectively termed protein quality control (PQC) systems [13]. Denatured proteins are recognized and then repaired by molecular chaperones, including heat shock proteins (HSPs) [12], while excessively denatured proteins undergo ubiquitination and are degraded through intracellular proteolytic machineries, including the ubiquitin-proteasome system (UPS) and autophagy [13]. Expression of HSPs induced by environmental or pathophysiological stress is largely regulated by heat shock factor 1 (HSF1), a transcriptional factor. Although HSF1 is sequestered in cytosol by binding with constitutive HSP90 under normal conditions, under cellular stress conditions, such as heat, hypoxia, and ethanol, HSP90 is recruited to denatured proteins, which cause a release of HSF1 to translocate to the nucleus and promote transcription of inducible HSPs. This process is known as heat shock response (HSR). Moreover, recent studies have revealed that HSF1 regulates various genes related to suppression of inflammation, as well as aging and cancer in stressed and non-stressed cells, indicating its importance as a master transcriptional factor in homeostasis [14–16]. In support of this notion, HSF1 knockdown has been reported to exacerbate pro-inflammatory responses *via* the activation of nuclear factor-κB (NF-κB) and activator protein-1 (AP-1) [17]. These transcriptional factors play important roles in inflammation and carcinogenesis, thus are considered to be promising targets for treatment of those pathologies [17]. Additionally, intracellular HSP70 induced by HSF1 has also been reported to possess an anti-inflammatory function in macrophages, while extracellular HSP70 plays a pro-inflammatory role [18].

Our previous study showed that zerumbone promotes ubiquitination and aggregation of cellular proteins in Hepa1c1c7 mouse hepatoma cells, which indicated its potential proteo-toxicity [11]. Furthermore, zerumbone has been shown to increase the expression of HSPs (HSP40, HSP70, HSP90) through HSF1 activation, and enhance the intracellular proteolysis mechanisms of UPS and autophagy, both of which are activated by zerumbone-induced proteo-stress for removing denatured proteins [10,11]. In the present study, we investigated whether HSF1 activation induced by zerumbone contributes to its anti-inflammatory functions in lipopolysaccharide (LPS)-stimulated RAW264.7 mouse macrophages. Interestingly, HSF1 down-regulation was found to significantly attenuate its suppressive effects on mRNA and protein expressions of iNOS and interleukin (IL)-1β. These results suggest that proteo-stress induced by zerumbone activates HSF1 for exhibiting its anti-inflammatory functions in macrophages.

## Materials and Methods

### Reagents

Dulbecco’s modified eagle medium (DMEM) and Opti-MEM^®^ were purchased from Life Technologies (Grand Island, NY), and fetal bovine serum (FBS) from Biological Industries (Beit HaEmek, Israel). Zerumbone was purified (>95%) as previously reported [1], briefly, fresh rhizomes of *Z*. *zerumbet* were extracted with methanol at room temperature and concentrated *in vacuo*. The aqueous extract was then partitioned between chloroform and deionized water to give a chloroform layer which was subjected to silica gel column chromatography (ethyl acetate/*n*-hexane, stepwise). Zerumbone in 5% ethyl acetate fraction was recrystallized from methanol Abs were obtained from the following sources: mouse anti-ubiquitin, rabbit anti-HSF1, and anti-rabbit horseradish peroxidase (HRP)-linked IgG Abs were from Cell Signaling Technology (Beverly, MA); mouse anti-HSP70 Ab was from Enzo Biochem Inc. (New York, NY); mouse anti-α-tubulin Ab was from EMD Bioscience (La Jolla, CA); goat anti-COX-2, rabbit anti-iNOS, and goat anti-lamin B Abs were from Santa Cruz Biotechnology (Santa Cruz, CA); and HRP-conjugated anti-mouse and HRP-conjugated anti-goat Abs were from DAKO (Tokyo, Japan). siRNAs and Lipofectamine^TM^ RNAiMAX were purchased from Invitrogen (Carlsbad, CA). LPS (*Salmonella enterica* serotype *typhimurium*) was purchased from Sigma-Aldrich (St. Louis, MO) and 4-phenyl butyric acid (PBA) from Cayman Chemical (Ann Arbor, MI). All other chemicals were purchased from Wako Pure Chemicals (Osaka, Japan), unless specified otherwise.

### Cell culture

RAW264.7 murine macrophages were purchased from the American Type Culture Collection (Rockville, MD). The cells were grown in DMEM supplemented with 10% heat-inactivated FBS and streptomycin (100 μg/mL) at 37°C in a humidified atmosphere of 95% air and 5% CO_2_.

### Western blot analysis

Cells were treated with the sample or vehicle [0.5% dimethyl sulfoxide (DMSO), *v/v*] for various times, then lysed in BioPlex cell lysis buffer (Bio-Rad Laboratories, Hercules, CA) (1% protease inhibitor cocktail, *v/v*). Proteins were detected using the appropriate specific primary Ab (1:2000), followed by the corresponding HOURP-conjugated secondary Ab (1:2000), as previously described [10].

### Filter trap assay

Filter trap assays were performed as previously reported [19]. Briefly, cells were seeded into 35-mm dishes and treated with the sample or vehicle for various times, then lysed in an ice-cold lysis buffer (20 mM Tris-HCl, pH 7.5; 137 mM NaCl; 1 mM ethylene glycol tetraacetic acid; 10% glycerol; 2% protease inhibitor cocktail; 1% protease inhibitor cocktail; 1% Tween 20, *v/v*) and collected by scraping. Samples were incubated on ice for 15 minutes, and then centrifuged for 10 minutes at 19,000 x *g* at 4°C. The chambers of a 96-well Dot-blot system (Bio-Dot® Microfiltration apparatus, Bio-Rad Laboratories, Hercules, CA) were covered with Immobilon-P membranes (upper) and filter paper (lower), which were pre-wetted with normalizing buffer, to establish a dot-blot system. After normalizing the samples to 1 μg/μL of protein with normalizing buffer (2% sodium dodecyl sulfate; 10 mM Tris-ethylenediaminetetraacetic acid, pH 7.5), each sample (100 μL) was loaded into the respective wells and vacuumed until all exited through the membrane. The membranes were subjected to western blot analysis, as described above, using anti-ubiquitin and anti-zerumbone-adducts Abs.

### Determination of nitrite concentration by Griess assay

The nitrite concentrations in media supernatants were determined by Griess reaction [20]. Cells were seeded into 96-well plates were treated with zerumbone or the vehicle for 1 hour before exposure to LPS (100 ng/mL) for 24 hours. Each supernatant was mixed with Griess reagent [1% sulfanilamide in 5% phosphoric acid, 0.1% *N*-(1-naphthyl) ethylenediamine dihydrochloride]. Absorbance of the reaction mixture was measured at 540 nm.

### Determination of the concentrations of TNF-α, IL-6, and IL-1β by enzyme-linked immunosorbent assay (ELISA)

Cells were seeded into 96-well plates, and treated with the sample or vehicle for 24 hours. Concentrations of tumor necrosis factor (TNF)-α, IL-6, and IL-1β in supernatants were quantified using an ELISA kit (eBioscience, San Diego, CA), according to the manufacturer’s instructions. The supernatants were diluted by 300- and 100-fold for TNF-α and IL-6, respectively, whereas there was no dilution for IL-1β.

### Nuclear-cytoplasmic fractionation

Cells were seeded into 35-mm dishes and treated with zerumbone or the vehicle for various times, or with heat at 43°C, then subjected to nuclear-cytoplasmic fractionation using an NE-PER Nuclear and Cytoplasmic Extraction Reagents Kit (Thermo Fisher Scientific, Wilmington, DE), according to the manufacture’s protocol. Each fraction was subjected to western blot analysis.

### RNA interference of HSF1

Transfection of siRNAs was performed using Lipofectamine^TM^ RNAiMAX, according to the manufacturer’s specifications. Briefly, after RAW264.7 cells were seeded into 24-well plates, each siRNA solution was added to Lipofectamine^TM^ RNAiMAX solution (1:25), then incubated for 20 min in serum-free Opti-MEM^®^. Then, this transfection mixture was diluted in serum-free Opti-MEM^®^ (final concentration of siRNA 10 nM), then cells were added and incubated for 6 hours. After replacing the medium with DMEM containing 10% FBS, the cells were incubated for another 24 hours.

### Quantitative real-time RT-PCR

Cells were seeded into 24-well plates, and treated with the sample or vehicle for 6 hours. Total RNA was isolated using TRIzol reagent (Invitrogen, Carlsbad, CA). The amount and purity of RNA were assessed by spectrophotometry using a SmartSpec® 3000 Spectrophotometer (BioRad Laboratories). cDNA was synthesized using 1 μg total RNA with an RNA PCR Kit (AMV). Thermal cycling was performed using a 7300 Real-Time PCR system with SYBR green PCR mix (Applied Biosystems, Foster City, CA). The PCR conditions were as follows: 30°C for 10 minutes, 55°C for 30 minutes, 95°C for 5 minutes, and 4°C for 15 minutes. The primer (Sigma-Aldrich) sequences are summarized in Table 1.

**Table 1:** Primers used for real-time RT-PCR

Gene	Primer	Sequence (5’-3’)
HSF1	Sense	ACTCCAACCTggACAACCTg
	Antisense	ggAggCTCTTgTggAgACAg
TNF-α	Sense	CTgTAgCCCACgTCgTAgC
	Antisense	TgggAgTAgACAAggTACAACCC
IL-6	Sense	TgCTggTgACAACAACAACggCC
	Antisense	gTACTCCAgAAgACCAgAgg
IL-1β	Sense	TTgACggACCCCAAAAgATg
	Antisense	AgAAggTgCTCATgTCCTCA
iNOS	Sense	AATCTTggAgCgAgTTgTgg
	Antisense	CAggAAgTAggTgAgggCTTg
COX-2	Sense	AgACCAggCACCAgACCAAAg
	Antisense	gCATTCTTTgCCCAgCACTT

### Statistical analysis

Each experiment was performed at least 3 times and values are shown as the mean±SE, as appropriate. Statistically significant differences between groups for each assay were determined using Student’s *t*-test. Differences were considered to be significant at *p* < 0.05.

## Results

### Induction of proteo-stress by zerumbone in RAW264.7

**Fig 1:**
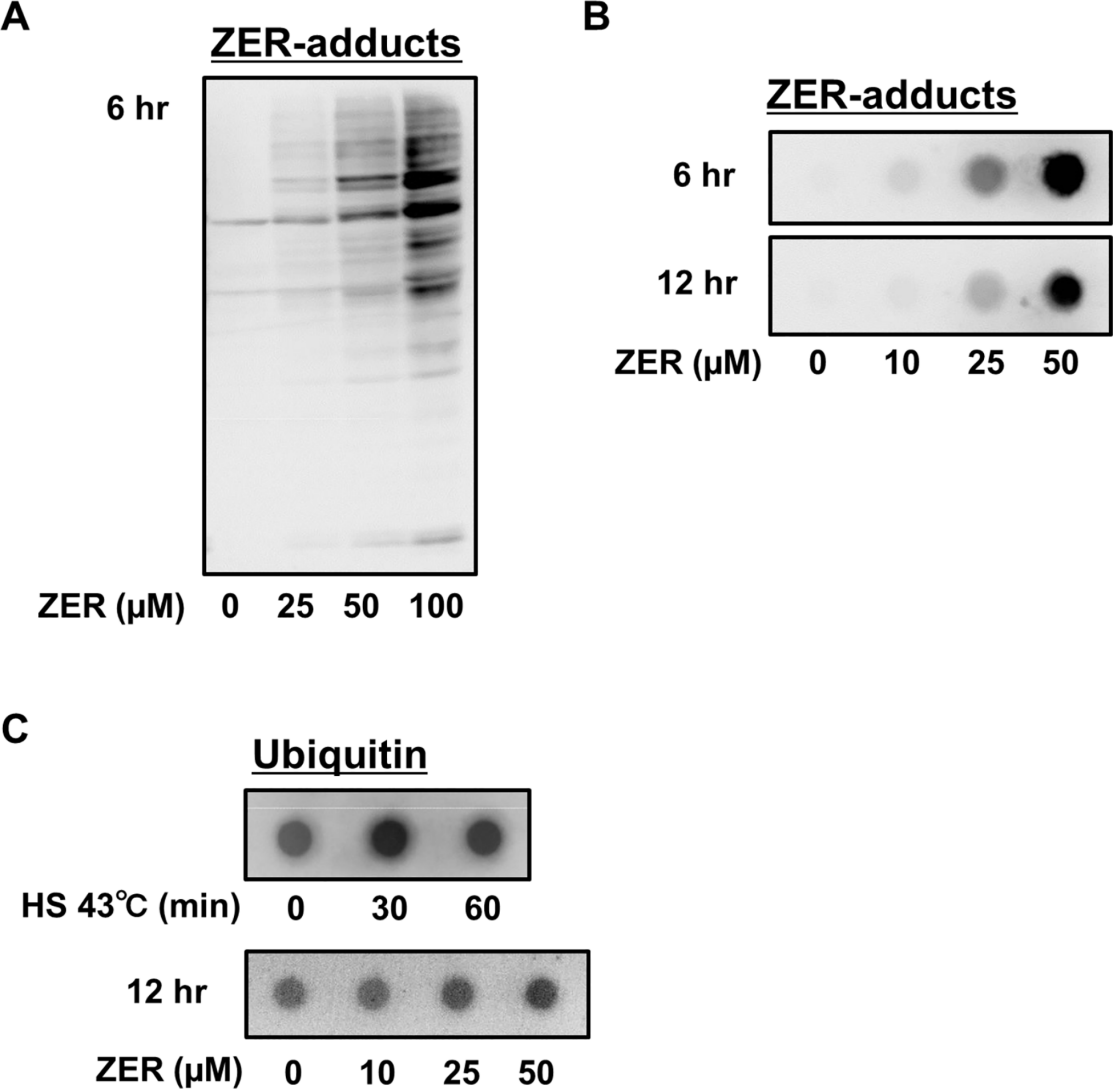
Zerumbone and HS induced proteo-stress. (A) RAW264.7 cells were treated with zerumbone (0–100 μM) for 6 hr, then lysed for western blot analysis using anti-zerumbone-adducts Ab. (B) Cells were treated with zerumbone (0–50 μM) for 6 or 12 hr, then lysed for filter trap assay using anti-zerumbone-adducts Ab. (C) Cells were treated with HS (incubation at 43°C in water bath) for 0–60 min or zerumbone (0–50 μM for 12 hr, then lysed for filter trap assay using anti-ubiquitin Ab.

We recently reported that zerumbone non-specifically reacted with protein cysteine residues to form thiol ethers in Hepa1c1c7 mouse hepatocytes [10]. To confirm its reactivity to cellular proteins in RAW264.7 mouse macrophages, we treated those cells with the vehicle alone or zerumbone for 6 hours, then cell lysates were subjected to western blotting with an Ab against zerumbone-adducts. The amounts of zerumbone-modified proteins were markedly increased in a concentration-dependent manner (Fig 1A). Next, we performed a filter trap assay with that Ab to investigate whether zerumbone increases protein aggregates, and found that treatment with zerumbone for 6 or 12 hours dramatically increased protein aggregates containing zerumbone-adducts (Fig 1B). Since abnormal proteins generated in the cytoplasm are known to undergo ubiquitination for their degradation [13], we conducted a filter trap assay with an Ab against ubiquitin. As shown in Fig 1C, both heat shock (HS) (43°C for 0–60 minutes) and zerumbone (0–50 μM for 12 hours) increased the amounts of protein aggregates showing ubiquitination, suggesting that zerumbone induces proteo-stress.

### Contribution of proteo-stress induced by zerumbone to its anti-inflammatory functions

**Fig 2:**
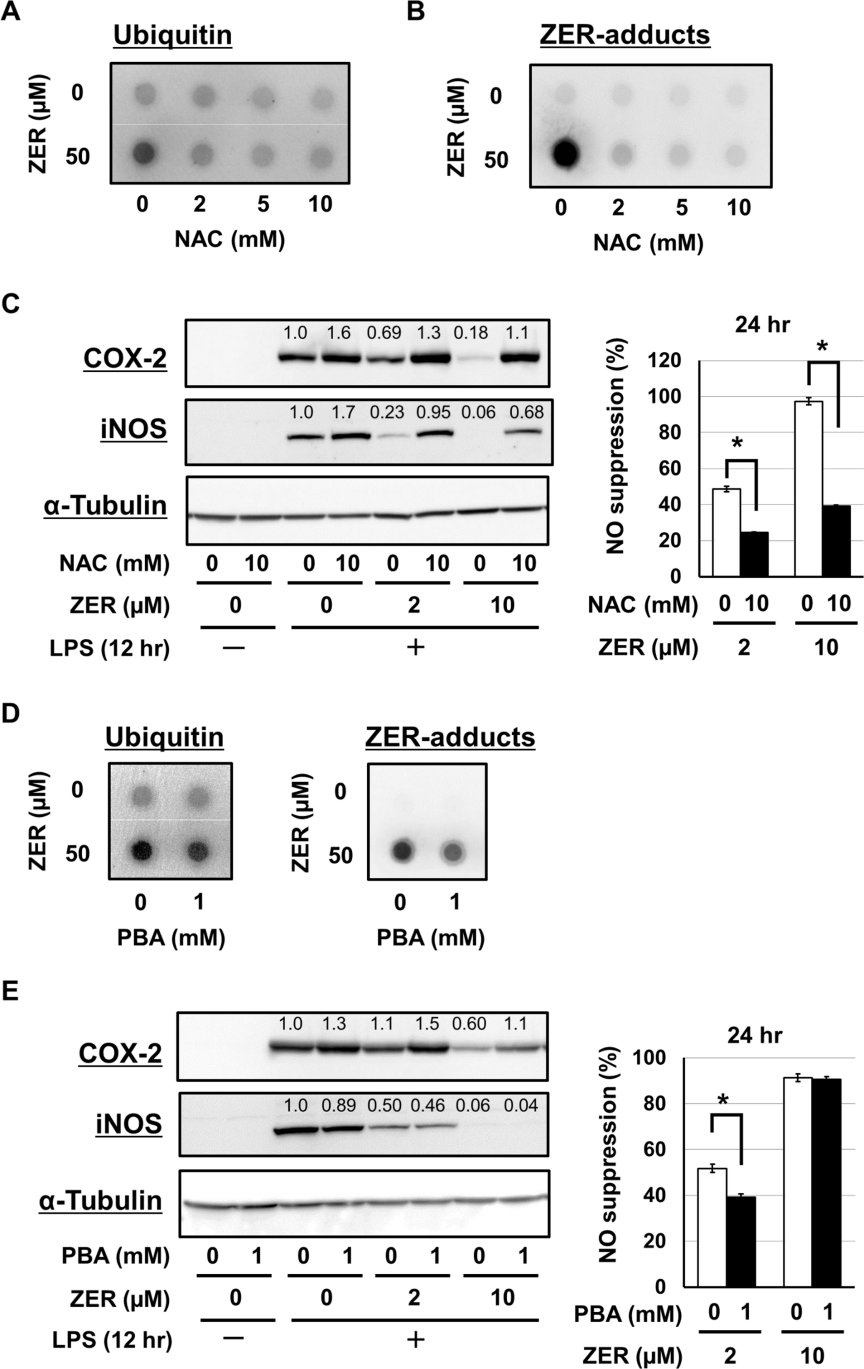
NAC and PBA attenuated both proteo-stress and anti-inflammatory functions of zerumbone. (A, B) RAW264.7 cells were pretreated with NAC (0–10 mM) for 1 hr. After incubation of the cells with zerumbone (0, 50 μM) for another 12 hr, cells were lysed for filter trap assay using anti-ubiquitin and anti-zerumbone-adducts Abs. (C) Cells were pretreated with NAC (0–10 mM) for 1 hr. After incubation of the cells with zerumbone (0–10 μM) for another 1 hr, cells were exposed to LPS (100 ng/mL) for 12 hr. Then, cells were subjected to western blot analysis using anti-COX-2, anti-iNOS, and anti-α-tubulin Abs. Also, after LPS stimulation for 24 hr, the supernatants were subjected to Griess assay. *P < 0.001. (D) Cells were pretreated with PBA (0, 1 mM) for 1 hr. After incubation of the cells with zerumbone (0, 50 μM) for another 12 hr, filter trap assay was done using anti-ubiquitin and anti-zerumbone-adducts Abs. (E) Cells were pretreated with PBA (0, 1 mM) for 1 hr. After incubation of the cells with zerumbone (0–10 μM) for another 1 hr, cells were exposed to LPS (100 ng/mL) for 12 hr. Then, cells were subjected to western blot analysis using anti-COX-2, anti-iNOS, and anti-α-tubulin Abs. Also, after LPS stimulation for 24 hr, the supernatants were subjected to Griess assay. *P < 0.01.

To ascertain the involvement of proteo-stress induced by zerumbone in its anti-inflammatory functions, we examined the suppressive effects of 2 different chemical reagents, *N*-acetyl-L-cysteine (NAC) and PBA on proteo-stress caused by zerumbone. NAC, an anti-oxidative nucleophile, possesses a reactive thiol group, thus we considered that pretreatment with an excess amount might competitively inhibit reactions between the thiol groups of proteins and zerumbone. We found that NAC notably inhibited protein aggregation induced by zerumbone, including ubiquitinated (Fig 2A) and zerumbone-adduct (Fig 2B) proteins. It is important to note that the suppressive effects of zerumbone on the expressions of COX-2 and iNOS protein, together with nitric oxide (NO) suppressive activities, were also markedly declined by pretreatment with NAC (Fig 2C).

PBA is an orally bioavailable chemical chaperone that has potential benefits as therapy for protein folding diseases, such as Huntington’s disease and Alzheimer’s disease [21–23], and has been reported to prevent protein aggregate accumulation and endoplasmic reticulum stress [24]. Interestingly, our results showed that pretreatment with PBA decreased protein aggregation induced by zerumbone (Fig 2D). In addition, this agent also attenuated the suppressive effects of zerumbone on COX-2 protein and NO generation, whereas it showed no effects on iNOS protein expression (Fig 2E).

### Promotion of nuclear translocation of HSF1 and increase of HSP70 expression by zerumbone

**Fig 3:**
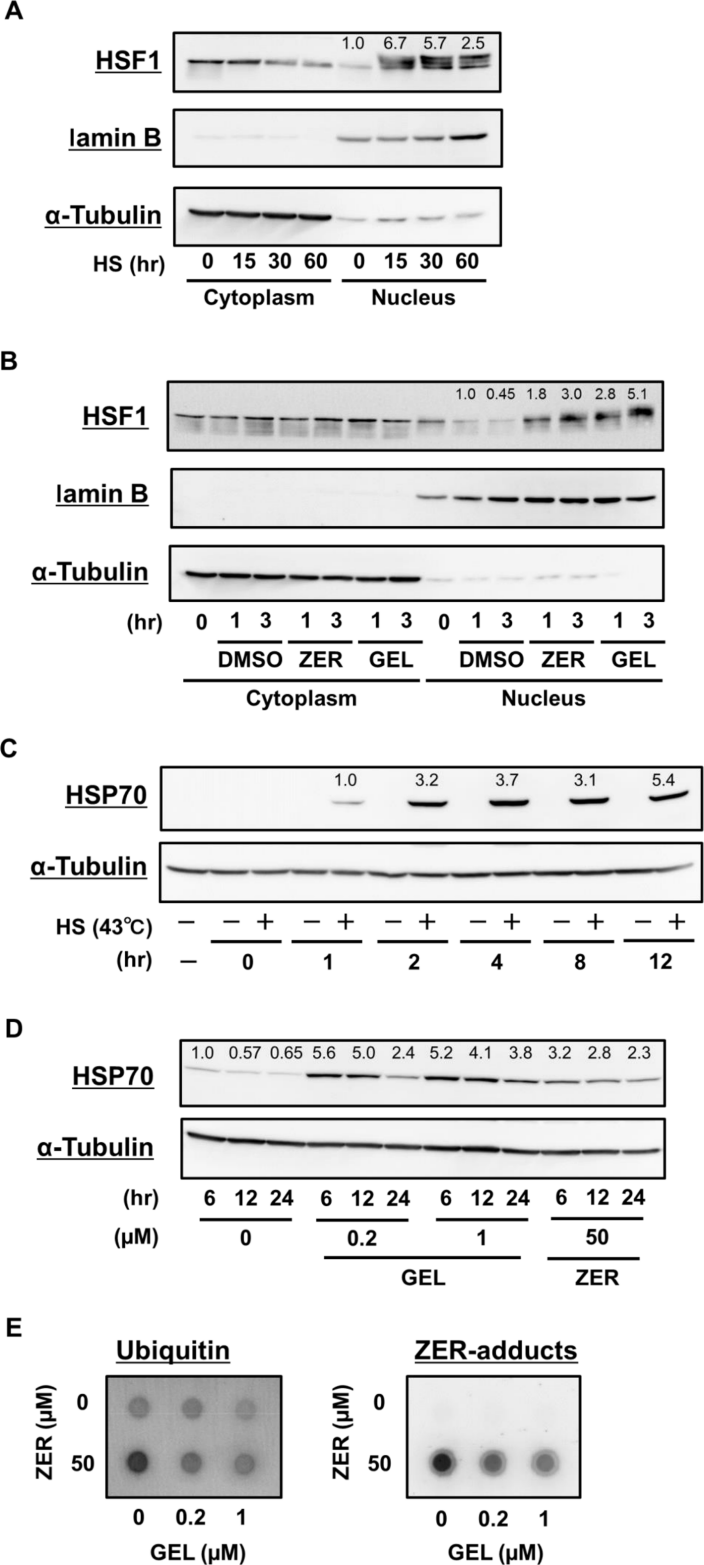
Zerumbone and HS promoted nuclear translocation of HSF1 and induced HSP70 expressions. (A, B) RAW264.7 cells were treated with the vehicle (DMSO), zerumbone (50 μM), or geldanamycin (1 μM) for 0–3 hr, then subjected to Nuclear-cytoplasmic fractionation. Cells were lysed for western blot analysis using anti-HSF1, anti-laminB (as a nucleus fraction marker), and anti-α-tubulin (cytoplasm) Abs. As a positive control, cells were treated with HS (incubation at 43°C in water bath) for 60 min. (C) Cells were treated with or without HS (incubation at 43°C in water bath) for 30 min. After recovery at 37°C for another 0–12 hr, cells were lysed for western blot analysis using anti-HSP70 and anti-α-tubulin Abs. (D) Cells were treated with vehicle (DMSO), zerumbone (50 μM), or geldanamycin (0.2, 1 μM) for 6–24 hr, then lysed for western blot analysis using anti-HSP70 and anti-α-tubulin Abs.

Transcriptional activation of HSF1 plays a key role in PQC systems by promoting HSR [25]. Translocation of HSF1 to the nucleus in response to HS or stress is tightly correlated with its phosphorylation, oligomerization, and DNA-binding activity [26]. As shown in Fig 3A, HS treatment promoted nuclear translocation of HSF1. Next, we examined whether zerumbone activates HSF1 in RAW264.7 cells. Zerumbone promoted nuclear translocation of HSF1 in a treatment time-dependent manner, with similar results seen for geldanamycin, a specific inhibitor of HSP90 and potent inducer of HSPs that functions through HSF1 transactivation, used as a positive control (Fig 3B). Following HSF1 translocation, treatment with HS, geldanamycin, and zerumbone up-regulated inducible HSP70 expression (Fig 3C and 3D).

### Anti-inflammatory functions by HSR inducers, HS and geldanamycin

**Fig 4:**
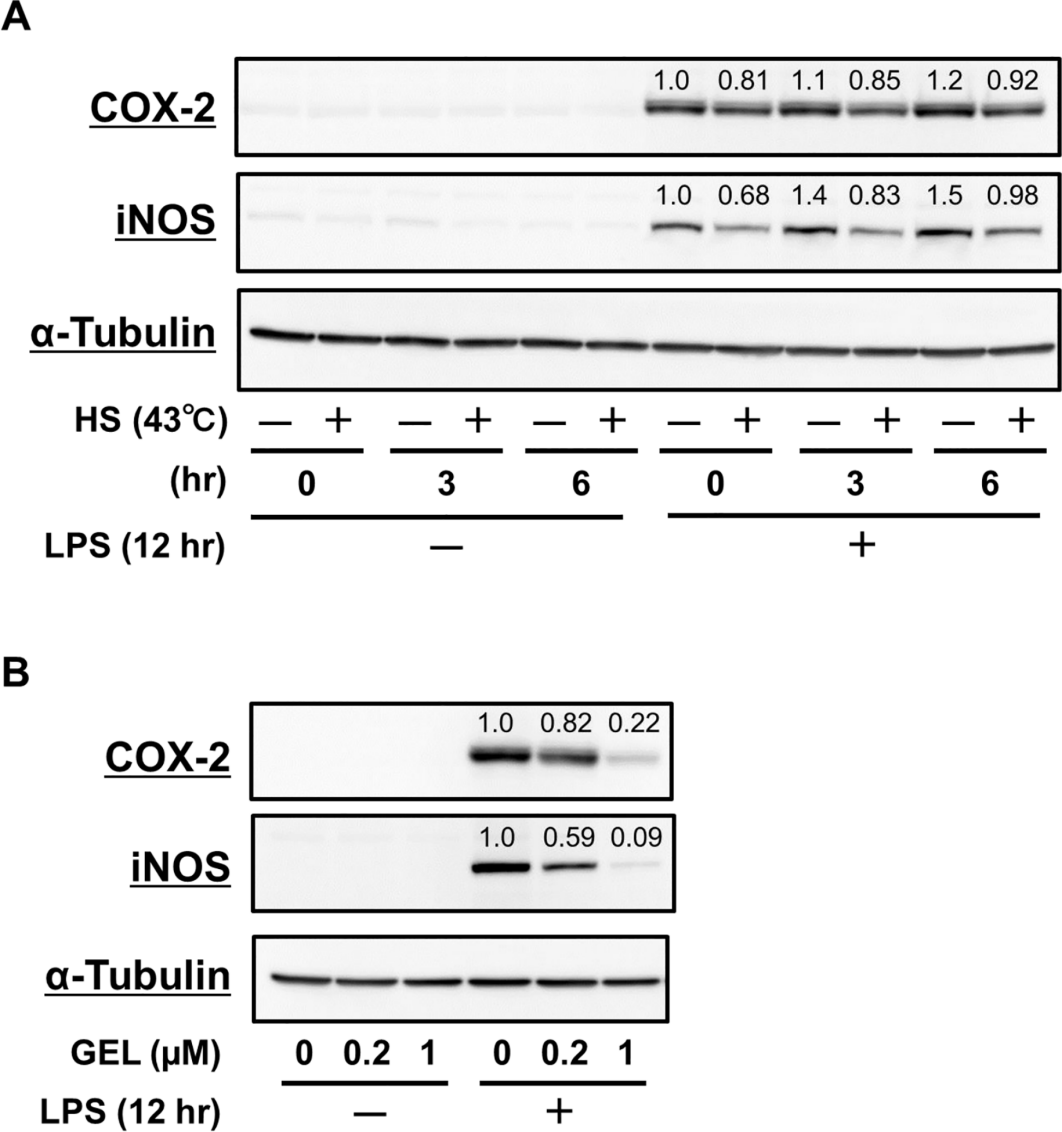
HS treatment and geldanamycin had anti-inflammatory functions. (A) RAW264.7 cells were treated with HS (incubation at 43°C in water bath) for 30 min. After recovery at 37°C for another 0–6 hr, cells were lysed for western blot analysis using anti-COX-2, anti-iNOS, and anti-α-tubulin Abs. (B) Cells were pretreated with geldanamycin (0–1 μM) for 6hr, then exposed to LPS (100 ng/mL) for 12 hr. Then, cells were lysed for western blot analysis using anti-COX-2, anti-iNOS, and anti-α-tubulin Abs.

Several reports have demonstrated that HSF1 activation and increment of expression of HSPs are related to suppression of pro-inflammatory responses [17,27], thus we explored the anti-inflammatory potential of HS and geldanamycin. HS treatment inhibited iNOS expression induced by LPS, while the effect on COX-2 was weak (Fig 4A). Furthermore, geldanamycin suppressed the expression of COX-2 and iNOS induced by LPS (Fig 4B), implying that HSF1 activation negatively regulates the expression of these pro-inflammatory mediators.

### Attenuation of the suppressive effects of zerumbone on iNOS and IL-1β induction by HSF1 down-regulation

**Fig 5:**
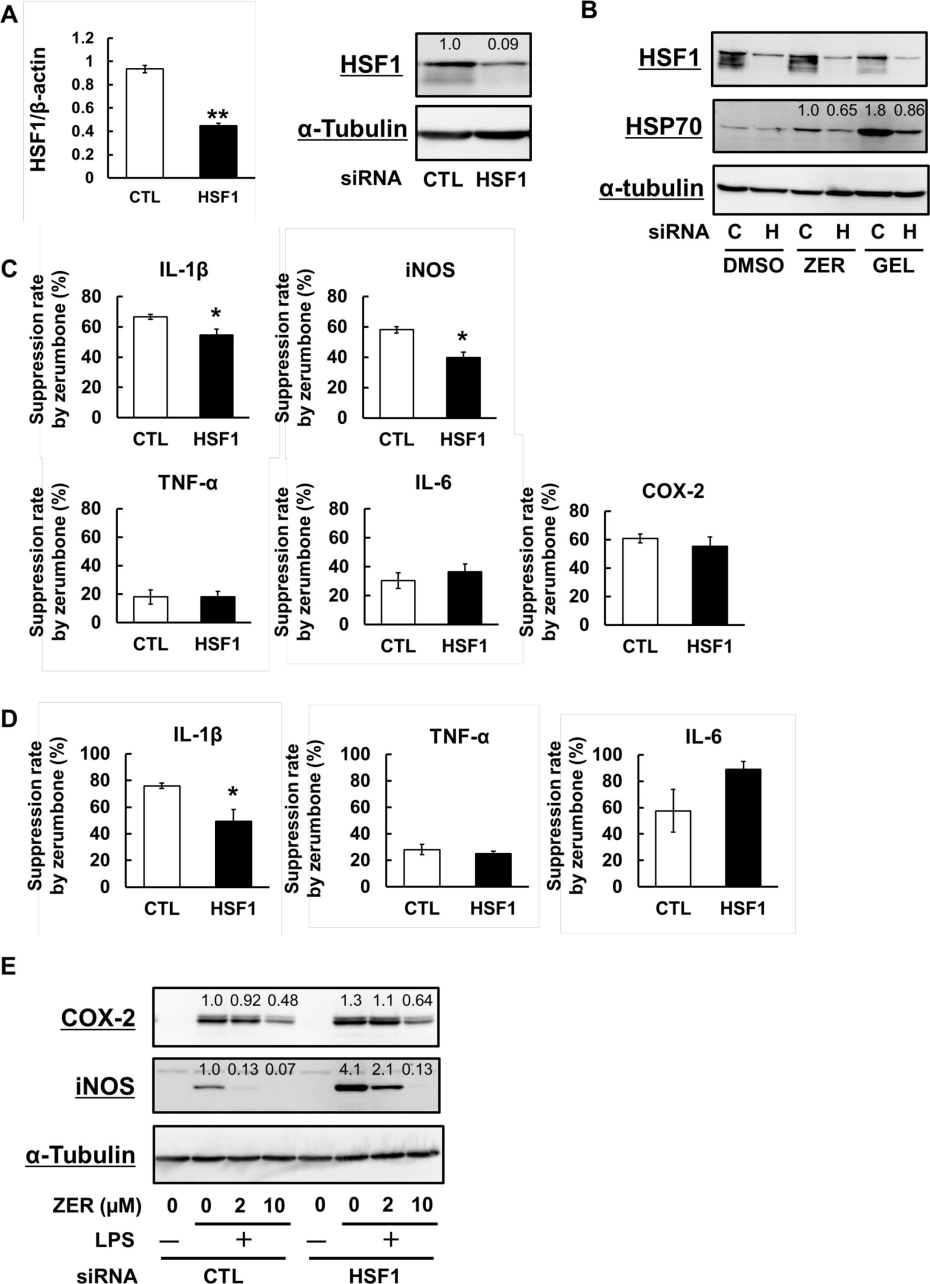
HSF1 down-regulation partially attenuated the anti-inflammatory effects of zerumbone. (A) Cells were treated with siRNA solution (control or HSF1 siRNA, 10 nM) for 6 hr. After recovery for another 24 hr, total RNA was subjected to qRT-PCR to semi-quantify the expression of HSF1 and β-actin, or cells were lysed for western blot analysis using anti-HSF1 and anti-α-tubulin Abs. (B) siRNA-transfected cells were pretreated with vehicle (DMSO), zerumbone (50 μM), or geldanamycin (1 μM) for 12 hr. Then, cells were lysed for western blot analysis using anti-HSF1, anti-HSP70, and anti-α-tubulin Abs. (C) siRNA-transfected cells were pretreated with zerumbone (0, 2 μM) for 1 hr. Exposed to LPS (100 ng/mL) for 6 hr, total RNA was subjected to qRT-PCR to semi-quantify the expression of TNF-α, IL-6, IL-1β, iNOS, and COX-2. (D) Exposed to LPS (100 ng/mL) for 24 hr, the concentrations of TNF-α, IL-6, and IL-1β in the culture media were determined by ELISA. (E) siRNA-transfected cells were pretreated with zerumbone (0–10 μM) for 1 hr, then exposed to LPS (100 ng/mL) for 12 hr. Cells were lysed for western blot analysis using anti-COX-2, anti-iNOS and anti-α-tubulin Abs. *P < 0.05, **P <0.005 versus CTL.

To investigate the importance of HSF1 for the anti-inflammatory functions of zerumbone, we down-regulated HSF1 expression using siRNA (Fig 5A), which resulted in attenuation of HSP70 induction by zerumbone and geldanamycin (Fig 5B). We then examined the effects of HSF1-knockdown on the expressions of pro-inflammatory genes induced by LPS, including TNF-α, IL-6, IL-1β, iNOS, and COX-2, which revealed that mRNA expressions of IL-6 and iNOS were notably increased by HSF1 knockdown (data not shown). Next, we pretreated with zerumbone 1 hour prior to LPS stimulation to investigate the effects of HSF1 down-regulation on the expressions of these pro-inflammatory genes. Although the suppression rates of mRNA expression of TNF-α, IL-6, and COX-2 by zerumbone were not altered in HSF1-knockdown cells, those of IL-1β and iNOS were significantly attenuated (Fig 5C). Similar to mRNA expressions, HSF1 down-regulation also affected extracellular secretion of IL-1β protein, but not of TNF-α or IL-6 (Fig 5D). Along a similar line, HSF1 down-regulation decreased the suppressive activities of zerumbone on the expressions of iNOS and COX-2 (Fig 5E).

## Discussion

Although diverse bioactivities of zerumbone have been reported [1–7], its mechanisms of action have not been fully uncovered. We recently found that zerumbone specifically targets Keap1 [2,8,9], while this agent binds to numerous proteins with low selectivity [10,11]. However, the role(s) of its non-specific interactions with cellular proteins remains to be clarified. Therefore, in the present study, we examined the possibility that proteo-toxic stress induced by zerumbone contributes to its anti-inflammatory functions.

Zerumbone increased protein aggregates in RAW264.7 cells, as indicated by filter trap assay results (Fig 1). Furthermore, it promoted translocation of HSF1 to the nucleus and increased protein expressions of HSP70, which were also shown by HS treatment (Fig 3). These findings indicate that zerumbone possesses a property to denature cellular proteins, which may induce HSR [10]. Interestingly, protein aggregates in cells treated with zerumbone for 12 hours were less abundant as compared to after 6 hours (Fig 1B), implying activation of degradation systems against intracellular abnormal proteins, such as UPS and autophagy. In support of this notion, we previously showed that zerumbone up-regulated the expressions of the 20S proteasome subunit β5 and pro-autophagic gene p62 in Hepa1c1c mouse hepatocytes [11]. To evaluate the involvement of proteo-stress induced by zerumbone in its anti-inflammatory functions, we employed 2 different reagents that are considered to mitigate proteo-stress. NAC, used to prevent zerumbone from reacting with protein thiols in a competitive manner, dramatically suppressed increment of protein aggregates, and attenuated its suppressive activities toward iNOS and COX-2 expressions and NO generation (Fig 2A–2C). However, NAC may also interfere with zerumbone to react with Keap1, which is known to possess highly reactive thiol groups [28], to attenuate Nrf2 activation by the agent. In addition, PBA, a reagent known to mitigate proteo-stress without disturbing thiol modification, was used. Molecular chaperones are major players in the PQC system and HSPs are their endogenous proteins, while there are also low molecular-weight compounds termed chemical chaperones [24]. PBA is a chemical chaperone that reduces accumulation of protein aggregates *in vivo* and *in vitro* and its hydrophobic moiety has been reported to interact with exposed hydrophobic segments of unfolded protein to prevent aggregation [24]. This agent has also been shown to be beneficial when used as the therapy for various proteo-toxic diseases, such as Alzheimer’s disease, Huntington’s disease, and Parkinson’s disease [21–23,29]. Interestingly, PBA treatment decreased protein aggregation induced by zerumbone and attenuated its suppressive activity toward COX-2 protein expression, whereas iNOS expression was not altered (Fig 2D and 2E). Collectively, our results suggest that proteo-stress induced by zerumbone partially contributes to its anti-inflammatory effects.

A number of studies have demonstrated that HSP overexpression may be a promising approach for treatment of neurodegenerative [30] and inflammatory [31] diseases. Geldanamycin, a naturally occurring benzoquinone ansamycin that selectively binds to and inhibits HSP90 to trigger HSR [32], has been demonstrated to suppress aggregation *via* induction of molecular chaperones in various neurodegenerative diseases [33–35]. Interestingly, we found that geldanamycin suppressed the protein expressions of COX-2 and iNOS in LPS-stimulated RAW264.7 cells (Fig 4B). Kacimi *et al*. reported that HSP70 up-regulation induced by HSP90 binding agents including geldanamycin exerted neuroprotective and anti-inflammatory effects in BV2 mouse microglia cells, and also noted that HSP90 inhibition by subsequent HSP70 induction blocked the anti-inflammatory effects of these agents induced by NF-κB [36]. Thus, the suppressive effects of geldanamycin on COX-2 and iNOS protein expressions (Fig 4B) may be associated with its HSP70 induction activity (Fig 3D).

HSR activation is orchestrated by HSF1, which transcripts *hsp* genes and rapidly induces their expression in response to various types of stress [37]. In non-stressed conditions, HSF1 is maintained in an inactive state by a direct association with HSP90. When cells are subjected to HS or other proteo-toxic stress, the resulting increment of protein misfolding leads to HSP90 dissociation from HSF1, which allows HSF1 activation through its trimerization, phosphorylation, nuclear localization, and DNA-binding [15,16]. Present findings showed that zerumbone promotes nuclear translocation of HSF1 in a time-dependent manner (Fig 3B), suggesting that this agent is capable of activating HSF1. In support of that notion, we recently found that zerumbone induced HSF1 phosphorylation at Ser326 in a different cell line [10]. HSF1 plays a pivotal role in suppression of pro-inflammatory cytokine genes, including TNF-α [38], IL-6 [39], and IL-1β [40], and our findings indicate that down-regulation of HSF1 leads to a marked decrease in anti-inflammatory activity by zerumbone (Fig 5C–5E). Furthermore, Chen *et al*. reported that HSF1 down-regulation exacerbated pro-inflammatory activation of NF-κB and AP-1 in vascular smooth muscle cells [17]. Also, in other studies, HSF1 repressed LPS-induced transcription of the *TNF-α* and *IL-1β* genes *via* its direct interaction with the *TNF-α*5’-untranslated region and nuclear factor of IL-6 respectively [38,40]. Furthermore, Takii *et al*. reported that HSF1 induced expression of activating transcriptional factor (ATF) 3, which negatively regulated IL-6 [39], and also showed that heat pretreatment inhibited LPS-induced expression of 86% of the genes examined, and that HSF1-induced ATF3 activation played important roles in iNOS and IL-6 suppressive activities following heat pretreatment [39]. Intriguingly, we previously reported that zerumbone did not affect the NFκB activity in LPS-stimulated RAW264.7 macrophages [7]. Together, the present results and these findings suggest a significant role of proteo-stress by zerumbone to exhibit its anti-inflammatory effects.

Recently, HSPs have attracted attention from researchers because of their therapeutic potential for patients with neurodegenerative and inflammatory diseases [30,31,41]. In this study, we showed that moderate proteo-stress induced by zerumbone activates HSR, thereby contributing to its anti-inflammatory functions. Interestingly, Muralidharan *et al*. reported that moderate alcohol-induced activation of HSF1 and induction of HSP70 inhibited pro-inflammatory cytokines resulting in endotoxin tolerance [42]. It is well known that alcohol possesses a property to denature proteins, which may be related to alcohol-induced HSF1 activation. In addition to HSP expression, other PQC systems, including UPS and autophagy, may contribute to the anti-inflammatory functions of zerumbone. Yao *et al*. reported that arctigenin, a natural dibenzylbutyrolactone, promoted degradation of iNOS through proteasomes, in which the carboxyl terminus of Hsc70-interecting protein (CHIP) played an important role [43]. CHIP, an E3 ubiquitin ligase, associates with iNOS and induces its ubiquitination and degradation *via* proteasomes [44], while it also promotes ubiquitination of unfolded proteins [45], which implies important roles of protein degradation systems in anti-inflammatory activities of zerumbone. In our previous study, we showed that zerumbone increased CHIP-dependent protein ubiquitination in mouse hepatocytes [11], suggesting that CHIP may contribute to the iNOS suppressive activity of zerumbone, though additional findings supporting this speculation are needed.

## Conclusions

**Fig 6:**
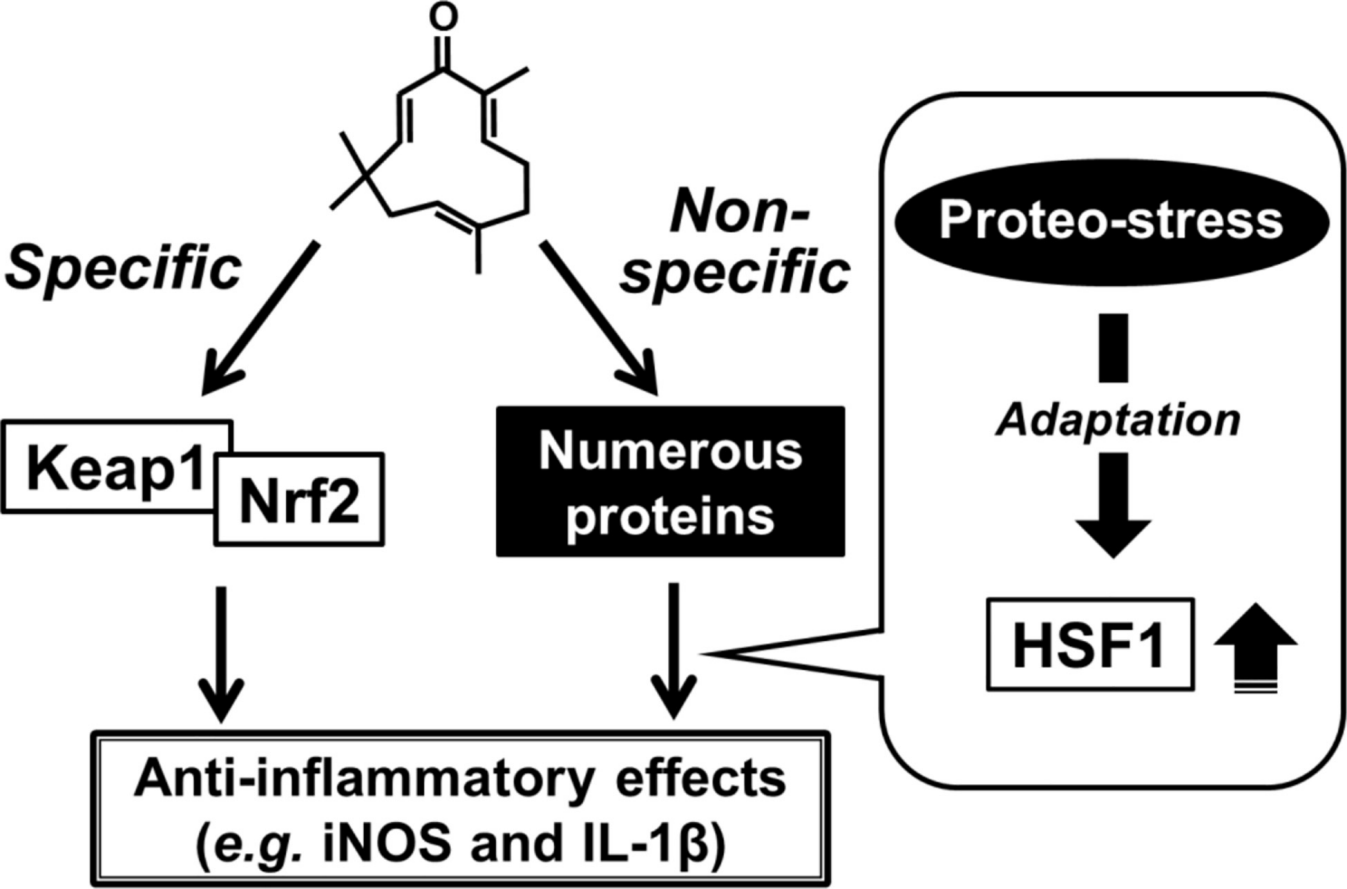
Proposed mechanisms of biological functions by zerumbone. Zerumbone may specifically bind to Keap1 and activates Nrf2, whose activation plays an important role in its detoxification and anti-oxidation [8, 2]. In addition to this mode of action, zerumbone is bound to various cellular proteins with low selectivity. Such non-specific interaction can induce proteo-stress and subsequently activate HSF1, which was found to partially contribute to anti-inflammatory functions of zerumbone.

The present findings showed that proteo-stress induced by zerumbone activates HSF1 for exhibiting its anti-inflammatory functions as well as other activities (Fig 6). In addition to this unique mode of action, it is also important to note that the specific interaction of zerumbone with Keap1 plays a significant role in its anti-inflammatory functions [8]. Various reports have shown that several phytochemicals induce HSPs [46,47], thus HSR induction by phytochemicals may partially account for the mechanisms underlying their anti-inflammatory functions. This mode of action is distinct from those of molecular-targeted drugs.

Because bioavailability of zerumbone has been uncovered to be poor in a mouse model (Murakami *et al*., unpublished data), its target site may be the gastrointestinal tract. This notion is supported by our previous work showing that zerumbone effectively prevented inflammation-associated colon carcinogenesis in mice [5]. Regarding the toxicity of zerumbone, it should be noted that Rahman *et al*. suggested that this agent has no apparent acute toxicity because the 50% lethal dose of zerumbone-loaded nanostructure lipid carrier was found to be higher than 200 mg/kg in BALB/c mice [48], although chronic toxicity should be examined in the future. Collectively, clinical use of this phytochemical for the regulation of inflammation-associated diseases in the gastrointestinal tract may warrant further investigation.

## Supporting Information

S1 Data All data of this work are available at https://figshare.com/s/bccc935c34bdd07fd073. (DOCX)
