# Supplementary material for: Possible Contribution of Zerumbone-Induced Proteo-Stress to Its Anti-Inflammatory Functions via the Activation of Heat Shock Factor 1
Source: PLoS One. 2016 Aug 18;11(8):e0161282. doi: 10.1371/journal.pone.0161282 (PMC4990220; doi:10.1371/journal.pone.0161282)
Supplement: S1 Data — (DOCX) [file pone.0161282.s001.docx]

**Supplemental Information 1**

We uploaded basic data to a Figshare site as follows. https://figshare.com/s/bccc935c34bdd07fd073
